# TCA-cycle metabolites in the nucleus: drivers of chromatin and epigenetic control

**DOI:** 10.1186/s12915-025-02423-4

**Published:** 2025-10-21

**Authors:** Serena Ghisletti, Marta Russo

**Affiliations:** 1 Department of Experimental Oncology, European Institute of Oncology (IEO) IRCCS, Milan, Italy; 2 DiSFeB, Department of Pharmacological and Biomolecular Sciences, Università Degli Studi Di Milano, Milan, Italy; 3 Biomedical Center (BMC), Division of Physiological Chemistry, Faculty of Medicine, LMU Munich, Munich, Germany

**Keywords:** Metabolism, Mitochondrial enzymes, Transcriptional regulation, Histone modifications

## Abstract

Mitochondrial enzymes are increasingly recognized for their ability to translocate to the nucleus, where they generate metabolites essential for epigenetic regulation and gene expression. Yet, whether this phenomenon broadly involves metabolic enzymes or is restricted to specific subunits remains unclear. In this review, we assess current evidence, highlight knowledge gaps, and suggest future directions on the nuclear localization and functions of metabolic enzymes, with a focus on acyl-CoA producers. Emerging studies reveal multiple mechanisms guiding these enzymes to chromatin for localized metabolite synthesis. Key questions concern nuclear import machinery, chromatin interactions, and the regulatory impact of their activity.

## Introduction

Beyond its role as a genetic information hub that regulates gene expression, the cell nucleus is also a site for localized metabolic activity, enabled by the presence in the nucleoplasm of specific enzymes involved in the synthesis of key metabolites. While traditionally associated with cytoplasmic and mitochondrial pathways, several metabolic enzymes have been found in the nucleus, where they contribute to chromatin remodeling and epigenetic modifications [1–5].

It was long assumed that nuclear metabolites and their precursors diffused passively from the cytoplasm or mitochondria. However, this diffusion-based model has recently been challenged by studies on intracellular molecular crowding, phase separation, and reaction–diffusion dynamics, which suggest a more controlled and compartmentalized regulation of nuclear metabolite availability [6]. Recent findings reveal that multiple tricarboxylic acid cycle (TCA) enzymes—including pyruvate dehydrogenase (PDH), oxoglutarate dehydrogenase (OGDH), and fumarate hydratase (FH)—generate metabolites directly in the nucleus, ensuring their rapid availability for nuclear processes [7–11].

Despite these discoveries, the precise mechanisms guiding nuclear recruitment of metabolism-related enzymes and their interactions with chromatin remain largely unknown. Some studies propose active transport mechanisms or interactions with nuclear structures, yet many questions remain unanswered. In this review, we will highlight recent advancements in understanding the nuclear localization and function of mitochondrial enzymes, with a particular focus on those involved in acyl-CoA production and their role in chromatin regulation.

## From mitochondria to the nucleus: the journey and function of acyl-CoAs

Acyl-CoAs are metabolites formed by the covalent linkage of an acyl group to coenzyme A via a thioester bond. These molecules are central to cellular metabolism and play a crucial role in nuclear processes, particularly in regulating gene expression. As donors for histone and non-histone protein acylation, acyl-CoAs influence nuclear signaling and transcriptional activity. Histone acylation includes various post-translational modifications such as acetylation and succinylation, which contribute to chromatin structure and transcriptional regulation [12–17].

Histone acetylation is the most well-characterized chromatin modification and depends on acetyl-CoA derived from glucose metabolism, fatty acid oxidation, and amino acid catabolism [18, 19]. Protein acetylation occurs co-translationally, regulating protein stability and localization [20], or post-translationally on lysine residues, modulating chromatin accessibility [21–24]. In histones, it weakens DNA interactions, promoting chromatin relaxation and transcription. Importantly, acetyl-CoA fluctuations directly impact histone acetylation, linking metabolism to chromatin remodeling [15, 16, 25–30].

**Fig. 1:**
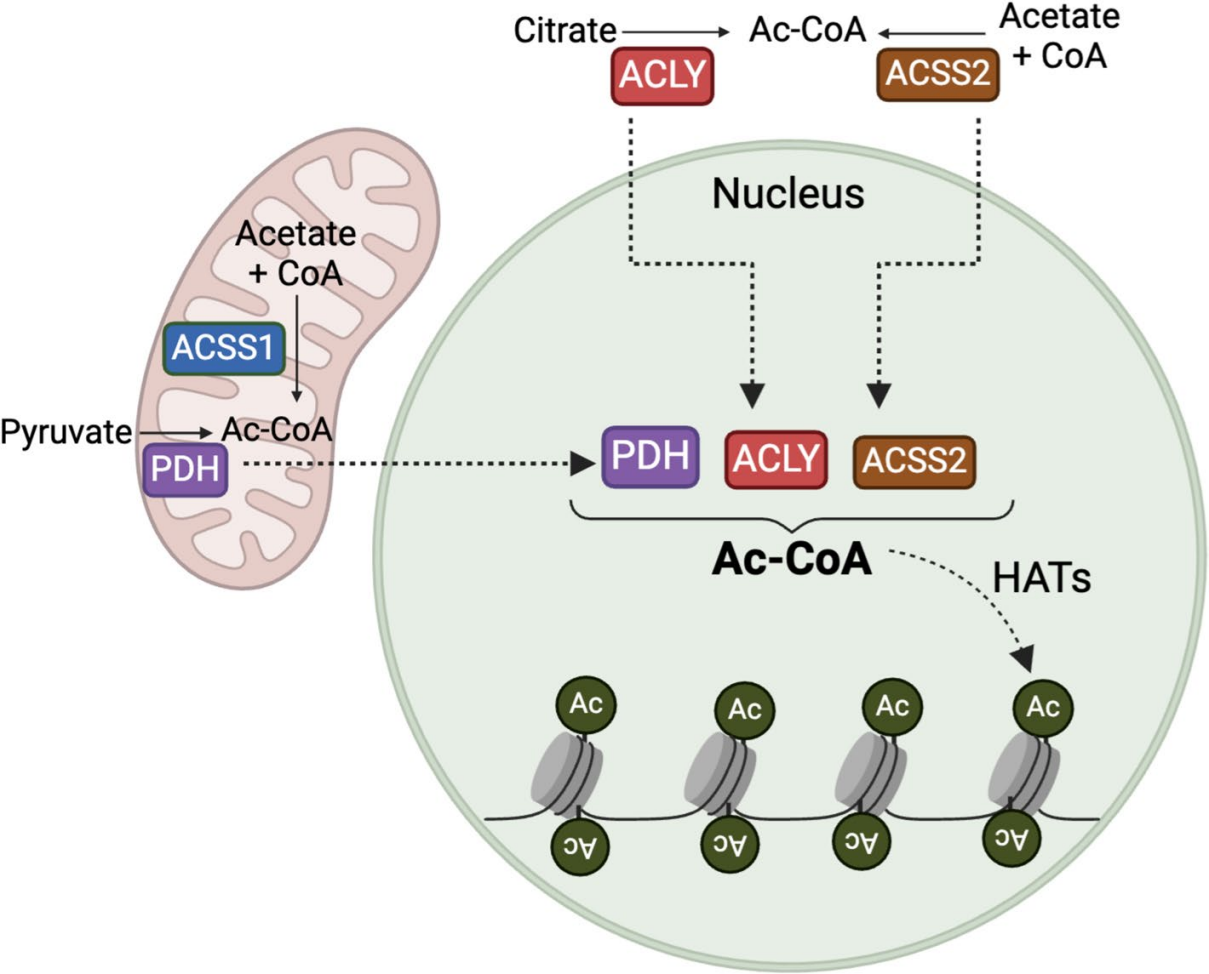
Translocation of acetyl-CoA producing enzymes to the nucleus. Acetyl-CoA is synthesized in the mitochondria by pyruvate dehydrogenase (PDH) from pyruvate and by acyl-CoA synthetase short-chain family member 1 (ACSS1) from acetate. In the cytoplasm, ATP-citrate lyase (ACLY) and acyl-CoA synthetase short-chain family member 2 (ACCS2) generate acetyl-CoA from citrate and acetate, respectively. PDH, ACLY, and ACCS2 are capable of translocating to the nucleus, where they locally produce acetyl-CoA, which plays a crucial role in nuclear functions, including histone acetylation

However, the interplay between acetyl metabolism and histone acetylation is influenced by intracellular compartmentalization [15, 18]. Acetyl-CoA is predominantly produced in mitochondria by two key enzymes: the PDH complex, a multienzyme system that irreversibly converts pyruvate into acetyl-CoA while linking glycolysis and glutaminolysis to the TCA cycle, and acyl-CoA synthetase short-chain family member 1 (ACSS1), which activates acetate to acetyl-CoA and thereby enables the utilization of acetate as a mitochondrial carbon source [18] (Fig. 1). However, mitochondrial membranes are impermeable to acetyl-CoA, meaning that the mitochondrial pool of acetyl-CoA cannot be directly utilized in the nucleus, where it is crucial for histone acetylation and other nuclear processes. Additionally, acetyl-CoA is a high-energy, unstable molecule, releasing 31.4 kJ/mol upon thioester bond hydrolysis—more than ATP hydrolysis. This instability requires local production near its sites of use.

Since mitochondrial acetyl-CoA cannot cross the mitochondrial membrane, it is exported as citrate or acetate, which are then converted back into acetyl-CoA in the cytoplasm or nucleus [31]. The two key enzymes responsible for this conversion are ATP-citrate lyase (ACLY) [12, 32–36], which cleaves citrate into acetyl-CoA and oxaloacetate to link mitochondrial export with cytosolic and nuclear acetyl-CoA supply, and acyl-CoA synthetase short-chain family member 2 (ACSS2) [37], which activates acetate to acetyl-CoA, similarly to the mitochondrial ACSS1. Both enzymes localize to the cytoplasm and nucleus (Fig. 1), and while acetyl-CoA is theoretically in equilibrium between the cytoplasm and nucleus due to its small size and passive diffusion, nuclear-localized production provides a crucial source for regulatory processes. Moreover, it is worth to mention that mammals also express a third isoform of the acyl-CoA synthetase short-chain family, ACSS3, which localizes to the mitochondrial matrix [32]. Unlike ACSS1 and ACSS2, ACSS3 preferentially uses propionate as a substrate and functions as a propionyl-CoA synthetase in the liver, thereby linking short-chain fatty acid metabolism to mitochondrial CoA pools.

Importantly, in addition to its canonical role in generating mitochondrial acetyl-CoA, the PDH complex has also been detected in the nucleus, where it contributes directly to nuclear acetyl-CoA availability and supports chromatin-associated metabolic processes [7, 9, 10, 38–40] (Fig. 1). In the nucleus, these enzymes establish subnuclear metabolic niches that locally enhance acetyl-CoA availability, underscoring the direct metabolic control of gene expression and other nuclear transactions.

## Nuclear ACLY: a central node in acetyl-CoA metabolism and chromatin regulation

ACLY, a homotetrameric enzyme, functions in both the cytoplasm and nucleus [33, 34]. It catalyzes the ATP-dependent conversion of mitochondria-derived citrate, exported via SLC25A1, into acetyl-CoA and oxaloacetate [35]. Highly expressed in lipogenic tissues, it supports de novo lipid biosynthesis and its generated acetyl-CoA fuels histone and non-histone acetylation [33, 36]. Although cytoplasmic ACLY-derived acetyl-CoA diffuses into the nucleus, nuclear ACLY directly contributes to nuclear acetyl-CoA pools, regulating epigenetics.

Wellen et al. first identified nuclear ACLY in HCT116 cells. siRNA-mediated silencing reduced global histone acetylation, demonstrating its role as the primary nuclear acetyl-CoA source. Moreover, ACLY is necessary during growth factor stimulation and adipocyte differentiation and selectively regulates genes involved in glucose metabolism [37].

Nuclear ACLY is crucial for DNA damage repair. Sivanand et al. showed that ionizing radiation triggers ACLY phosphorylation at serine 455 (S455) via ataxia-telangiectasia mutated (ATM) and protein kinase B (AKT), enhancing homologous recombination (HR) by recruiting breast cancer type 1 susceptibility protein (BRCA1) to damage sites. ACLY loss disrupts BRCA1 localization, impairing repair and increasing genomic instability, particularly under poly (ADP-ribose) polymerase (PARP) inhibition, making ACLY-deficient cells hypersensitive to DNA damage [38].

SIRT6, a NAD + -dependent sirtuin, regulates nuclear ACLY to maintain genomic stability and suppress tumor progression. Zheng et al. found that SIRT6 loss increases nuclear ACLY accumulation, raising acetyl-CoA levels and histone acetylation, which enhances cell adhesion, migration, and tumor progression. Mechanistically, SIRT6 suppresses tumor growth through dual control of histone acetylation and ACLY nuclear localization [39].

Moreover, nuclear ACLY modulates immune responses. Santarsiero et al. showed that in activated macrophages, ACLY translocates to the nucleus and acetylates nuclear factor kappa B (NF-κB) subunit p65, enhancing its activation and promoting transcription of inflammatory genes like IL-1β and PTGS2. ChIP assays confirmed reduced NF-κB binding to these promoters upon ACLY inhibition, validated by sequential immunoprecipitation with anti-p50 and anti-AcK310 p65 antibodies [40].

Recently, Lazaropoulos et al. reported ACLY nuclear translocation in fibroblasts upon fibrotic stimulation, facilitating TGFβ-mediated myofibroblast activation by promoting H3K27ac at fibrotic gene loci. Co-immunoprecipitation showed that ACLY interacts with transcription factors SMAD2/3, recruiting it to pro-fibrotic genomic regions for local acetyl-CoA production [41]. In chemoresistance, Wang et al. found that ACLY interacts with NAT10, a nuclear acetyltransferase, which acetylates ACLY at K468, preventing its degradation via SQSTM1-mediated proteasomal pathways. This stabilization enhances nuclear acetyl-CoA levels, increasing H3K27ac at chemoresistance-related genes like CYP2C9 and PIK3R1 [42].

As a nuclear metabolic-epigenetic regulator, ACLY plays a critical role in cancer, fibrosis, immune activation, and DNA repair, making it a promising therapeutic target. Further research is needed to fully elucidate its disease-specific mechanisms and therapeutic potential.

## ACSS2 in the nucleus: linking acetate metabolism to gene regulation and disease

ACSS2 converts acetate and free CoA into acetyl-CoA [43, 44]. While primarily cytoplasmic, ACSS2 was among the first acetyl-CoA-producing enzymes identified in the nucleus, where it supports histone acetylation [45–47]. However, recent studies indicate that nuclear acetyl-CoA does not act as a global epigenetic modifier but is instead involved in the selective acetylation of specific nuclear targets, including histones and other regulatory proteins [40, 48–50]. For instance, nuclear ACSS2 facilitates the acetylation of hypoxia-inducible factor 2-alpha (HIF-2α) via CREB-binding protein (CBP), underscoring its role in oxygen-sensitive transcriptional regulation [51].

Nuclear ACSS2 has been also extensively studied in the brain, where acetate serves as a key carbon source, particularly under glucose-limited conditions. In neurons, ACSS2-generated acetyl-CoA directly modulates histone acetylation and spatial memory formation by recruiting CBP to specific chromatin loci [47]. Recent findings further highlight its essential function in fear memory formation [52]. ACSS2-deficient mice exhibit normal baseline behavior, but show pronounced deficits in long-term fear memory, linked to reduced histone acetylation and impaired expression of key memory-related genes in the dorsal hippocampus. Similarly, small-molecule ACSS2 inhibitors disrupt fear memory formation, emphasizing its therapeutic relevance in neurological disorders. These findings reveal a previously underappreciated role of nuclear ACSS2 in epigenetic regulation and behavioral responses in fear and anxiety-related contexts, suggesting new therapeutic avenues for treating anxiety disorders and cognitive impairments.

Although acetate is not a primary carbon source in normal mammalian cells, ACSS2 expression is significantly upregulated under specific metabolic conditions, particularly in cancer. In oxygen- and serum-deprived environments, nuclear ACSS2 levels rise, enabling tumor cells to metabolize acetate. ACSS2 facilitates acetate recycling by capturing acetate released during histone deacetylation for reuse by histone acetyltransferases. This mechanism is crucial in hypoxic, nutrient-deprived tumor regions, where ACSS2 sustains cancer cell survival and epigenetic adaptation [45].

Additionally, glucose deprivation in glioblastoma cells triggers the nuclear translocation of ACSS2, which subsequently activates lysosomal and autophagosomal gene expression. This occurs through the localized production of acetyl-CoA from acetate, which facilitates histone acetylation and transcriptional activation [46]. Interestingly, the Acs2 ortholog of ACCS2 in budding yeast has recently been identified as an integral subunit of the SESAME (Serine-Responsive SAM-Containing Metabolic Enzyme) complex. In this context, Acs2 regulates telomere silencing and influences cellular senescence. At the molecular level, Acs2, as part of the SESAME complex, promotes subtelomeric-specific histone acetylation. A similar mechanism is observed in mammalian cells, where acetate can increase subtelomeric acetylation and accelerate senescence [53].

A key question in nuclear ACSS2- and ACLY-mediated metabolism is how cancer cells sustain proliferation and histone acetylation when these enzymes are non-functional. Izzo et al. addressed this by generating ACLY- and ACSS2-deficient cancer cells. Surprisingly, despite the double knockout (DKO), cells remained viable, proliferated, and maintained a nuclear-cytosolic acetyl-CoA pool, sustaining protein acetylation. ^13^Carbon-tracing experiments revealed that fatty acids and glucose serve as alternative acetyl-CoA sources. Indeed, in glucose metabolism, acetyl-CoA production is partly mediated by the carnitine shuttle and carnitine acetyltransferase (CrAT). When ACLY is absent, acetyl groups are exported as acetylcarnitine, enabling cytosolic acetyl-CoA synthesis and de novo lipogenesis. Izzo and colleagues demonstrated that acetylcarnitine also enhances histone acetylation and cell proliferation in an ACLY- and ACSS2-deficient environment via lysine acetyltransferase (KAT) p300. These findings indicate that acetylcarnitine transport plays a key role in transferring mitochondrial acetyl-CoA to the cytosol, supporting histone acetylation, lipogenesis, and proliferation [54]. Further research is needed to determine whether the carnitine shuttle or acetylcarnitine uptake compensates for ACLY and ACSS2 loss in other contexts, such as adipose metabolism under a high-fat diet.

Previous studies have demonstrated a regulated interplay between ACLY and ACSS2, with compensatory mechanisms activated upon loss of one of these enzymes to mitigate drastic reductions in the cellular acetyl-CoA pool. However, these compensatory mechanisms do not appear to play major roles at the nuclear level in regulating chromatin dynamics. For example, in CD8⁺ T cells, ablation of ACLY activates an alternative, acetate-dependent pathway for acetyl-CoA production mediated by ACSS2. Nevertheless, chromatin accessibility in CD8⁺ T cells is predominantly regulated by ACLY, with only a small subset of loci dependent exclusively on ACSS2, even in the absence of ACLY [55]. Similarly, in a conditional mouse model of ACLY deficiency, ACSS2 expression is strongly upregulated [56]. Yet, consistent with earlier findings from RNAi-mediated ACLY silencing [37, 57], global levels of histone acetylation are markedly reduced following genetic deletion of ACLY, despite increased ACSS2 expression. Collectively, these results indicate that ACLY is the dominant supplier of acetyl-CoA for histone acetylation. In its absence, cells can utilize acetate to generate acetyl-CoA via ACSS2, but high exogenous acetate availability is required to restore histone acetylation to levels comparable to ACLY-proficient cells. Thus, ACSS2 provides only partial compensation for ACLY loss, suggesting that acetate-derived acetyl-CoA is not efficiently channeled into histone acetylation. This supports the hypothesis that acetylation of specific chromatin regions may be regulated not only by the corresponding acetyltransferases but also by distinct acetyl-CoA–producing enzymes.

More recently, to investigate acetate-dependent regulation of chromatin modifications, histone acetylation was analyzed in pancreatic ductal adenocarcinoma (PDAC) cells cultured under low-pH conditions [58]. PDAC-associated stromal cells secrete acetate through an ACLY-dependent mechanism. Within PDAC cells, ACSS2 channels this exogenous acetate to support dynamic remodeling of the cancer epigenome and transcriptome, thereby promoting cancer cell survival in an acidic microenvironment. Acetate supplementation enhanced the acetylation of several histone marks, including H3K9, H3K18, and H3K27, across multiple PDAC cell lines, demonstrating that acetate fosters cancer cell survival through the remodeling of histone modifications. These findings emphasize that acetyl-CoA metabolism, regulated by both ACLY and ACSS2, is a key determinant of histone acetylation levels and is functionally critical for the survival of pancreatic cancer cells under acidic stress.

Taken together, these observations indicate that the interplay between ACLY and ACSS2 in gene regulation is highly complex. Comprehensive, genome-wide analyses of their respective contributions to histone acetylation—as well as to the acetylation of non-histone protein substrates—will be required to fully elucidate their distinct and overlapping roles.

Recently, Willnow et al. highlighted ACSS2’s role in histone acetylation in the outer rim of the *Drosophila* wing imaginal disc, a model for spatial histone acetylation and metabolism in growing tissues. In *Drosophila*, histone acetylation is regulated by nuclear positioning, with nuclei migrating between apical and basal epithelial regions. Surface nuclei exhibit higher H3K18ac levels and contain more ACSS2, suggesting enhanced acetate-to-acetyl-CoA conversion. Cross-sectional analysis confirmed ACSS2 enrichment at the tissue surface, aligning with endogenous ACSS2 expression patterns. Fatty acid β-oxidation, the primary acetyl-CoA source in this region, is upregulated, and its inhibition reduces H3K18ac, particularly in genes linked to disc development [59]. The mechanism behind ACSS2’s specific localization remains unclear, requiring further investigation. Exploring whether similar histone acetylation patterns occur in other organisms or tumors—where tissue architecture and histone modifications are disrupted—may yield valuable insights.

## Nuclear localization of 2-ketoacid dehydrogenases: a paradigm shift

2-Ketoacid dehydrogenase complexes, once thought to be solely mitochondrial, are essential for the TCA cycle and metabolism. This enzyme family, including PDH, OGDH, and the branched-chain α-ketoacid dehydrogenase complex (BCKDH), converts key substrates into acetyl-CoA or succinyl-CoA. These complexes share a conserved structure, comprising E1 (dehydrogenase), E2 (dihydrolipoyl acyltransferase), and E3 (dihydrolipoamide dehydrogenase) [60–64]. Recent findings reveal PDH, OGDH, and BCKDH also localize in the nucleus, linking them to chromatin dynamics and gene regulation.

Sutendra et al. first identified nuclear PDH as an intact, active complex in mammalian nuclei. Its knockdown reduced de novo acetyl-CoA synthesis and histone acetylation. Nuclear PDH levels varied with the cell cycle, increasing with serum stimulation and mitochondrial stress. PDH inhibition impaired histone acetylation, G1-S transition, and S-phase marker expression, underscoring its role in cell cycle regulation [10].

Nagaraj and colleagues identified transient nuclear localization of mitochondrial TCA enzymes, including PDH, during early embryonic development. In mice, nuclear PDH peaked at the 2-cell stage, and in humans, at the 8-cell stage before exclusion at the morula stage. This nuclear PDH played a crucial role in epigenetic remodeling during zygotic genome activation (ZGA), highlighting its importance in early development [9].

Chen et al. identified nuclear PDH in prostate cancer cells, directly regulating lipid biosynthesis genes via histone acetylation. Nuclear PDH promoted sterol regulatory element-binding transcription factor (SREBF)-target gene expression, linking it to tumorigenesis and making it a potential therapeutic target [7].

Moreover, nuclear PDH translocation is linked to stress responses. Srivastava et al. studied anthracycline-induced cardiotoxicity in human iPSC-derived cardiomyocytes (hiPSC-CMs) and observed nuclear PDH-E1 translocation following doxorubicin, a chemotherapy drug causing cardiotoxicity. To assess its role, they engineered a lentiviral system with a nuclear localization signal (NLS) for all PDH subunits, confirming its catalytic activity via NAD + to NADH conversion in isolated nuclei. These findings suggest nuclear PDH aids cardiomyocyte stress adaptation [65].

In immune regulation, Mocholi et al. found that antigen-specific T cell receptor (TCR) engagement triggers PDH-E1 nuclear translocation in CD4 + T cells, promoting activation via extramitochondrial pyruvate-dependent acetyl-CoA production. PDH-E1 interacts with p300 acetyltransferase and H3K27ac, linking metabolism to chromatin remodeling. Proximity ligation assay (PLA) confirmed nuclear PDH-E1 localization and histone modifier interactions [66]. Similarly, Li and colleagues observed nuclear PDH-E1 in somatic cell reprogramming, primed-to-naive transition, and totipotency acquisition. Overexpressing nuclear PDH-E1 enhanced histone acetylation, confirmed by ChIP-seq, with increased p300 recruitment at pluripotency gene promoters (Sox2, Pou5f1, Nanog), suggesting nuclear acetyl-CoA fluctuations influence gene expression. However, both studies detected only PDH-E1 in the nucleus, not the full PDH complex. Since PDHA1 alone lacks catalytic function, its potential nuclear role remains uncertain and requires further investigation [67].

Recently, Zhang and colleagues showed that PDHE1α is recruited to chromatin via polyADP-ribosylation, generating acetyl-CoA to enhance chromatin acetylation and relaxation at double-strand breaks (DSBs). This supports efficient repair, genome stability, and cancer cell resistance to DNA damage. Blocking PDHE1α recruitment disrupts repair, increasing genome instability and radiosensitivity [68].

Beyond PDH, the OGDH complex also translocates to the nucleus. Wang et al. found that nuclear OGDH interacts with KAT2A/GCN5 at gene promoters, facilitating histone succinylation. Succinyl-CoA directly binds to KAT2A, catalyzing H3K79 succinylation near transcription start sites. Disrupting nuclear OGDH impairs gene expression, tumor proliferation, and growth, revealing a novel histone modification mechanism involving nuclear OGDH and KAT2A [11].

**Fig. 2:**
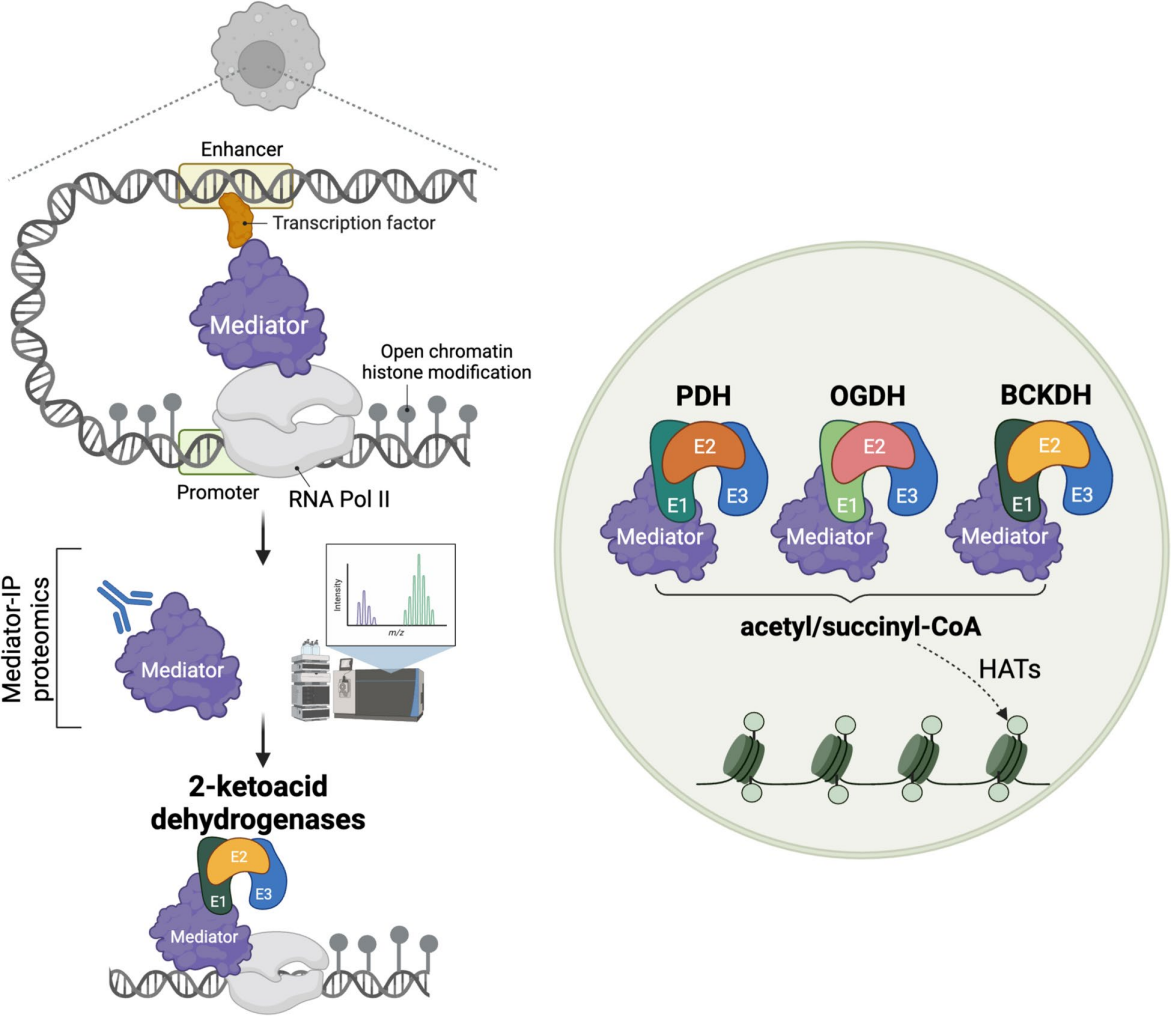
2-Ketoacid dehydrogenases are associated with the Mediator complex, providing a local supply of acetyl-CoA and succinyl-CoA. Left panel: Nuclear immunoprecipitation (IP) coupled with mass spectrometry (IP-MS) of purified nuclear extracts reveals that 2-ketoacid dehydrogenase holo-complexes are stably associated with the Mediator complex. Right panel: Pyruvate dehydrogenase (PDH), α-ketoglutarate dehydrogenase (OGDH), and branched-chain ketoacid dehydrogenase (BCKDH) are recruited to chromatin through the Mediator complex. These acyl-CoA-producing enzymes generate local supplies of acetyl-CoA and succinyl-CoA, which subsequently influence histone modifications

**Fig. 3:**
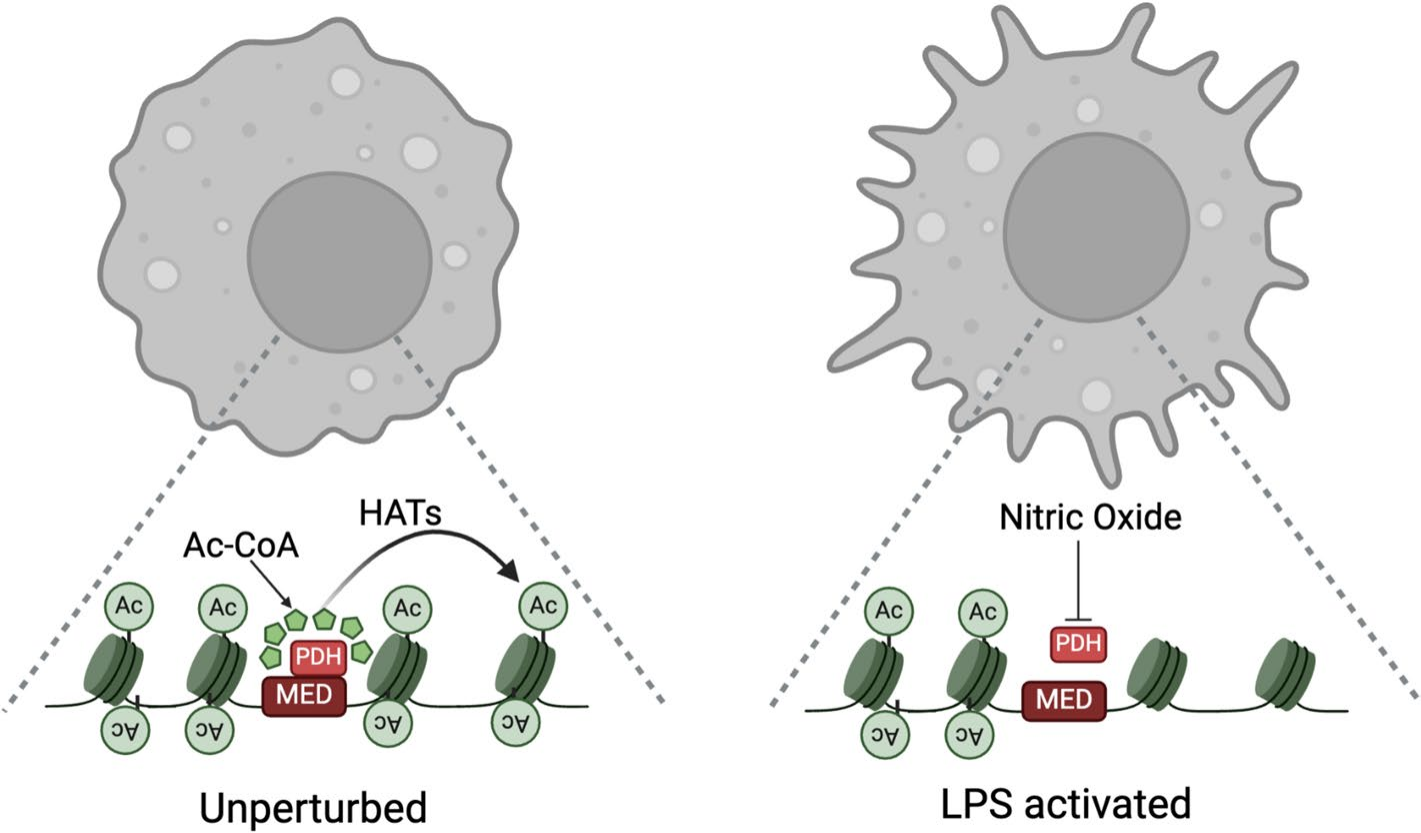
Regulation of PDH-mediated histone acetylation in macrophages. Left panel: In unperturbed macrophages, pyruvate dehydrogenase (PDH) associated with the Mediator complex provides a local supply of acetyl-CoA for histone acetylation. Right panel: Nitric oxide (NO) produced following prolonged exposure to the inflammatory stimulus lipopolysaccharide (LPS) inhibits PDH activity. This inhibition promotes the dissociation of PDH from the Mediator complex, impacting the de novo acetylation of regulatory genomic regions

Expanding on this, we recently identified 2-ketoacid dehydrogenases as RNA Polymerase II (Pol II) transcriptional interactors in macrophages [50]. Using nuclear IP-MS in purified nuclear extracts, we found PDH, OGDH, and BCKDH holo-complexes associated with the Mediator complex, a Pol II co-regulator (Fig. 2). ChIP-seq and super-resolution imaging confirmed strong genomic overlap in Mediator and 2-ketoacid dehydrogenase distributions. Enzymatic assays showed Mediator-bound PDH remains catalytically active, producing acetyl-CoA and NADH. H3K27Ac ChIP-seq revealed that this localized acetyl-CoA synthesis supports de novo histone acetylation at specific chromatin sites. Additionally, we found that during the late stage of LPS activation, when nitric oxide (NO) is produced, PDH becomes functionally inhibited and dissociates from the Mediator complex. As a result, PDH no longer contributes to histone acetylation under these conditions (Fig. 3). These findings establish PDH, OGDH, and BCKDH as chromatin-associated acetyl-CoA suppliers for histone acetylation, reinforcing their nuclear role in gene regulation.

These studies redefine 2-ketoacid dehydrogenases as nuclear metabolic regulators, extending their role beyond mitochondria. Their nuclear localization links acyl-CoA-producing enzymes to chromatin structure, transcription, cell cycle, embryonic development, inflammatory response, and tumor metabolism.

## From mitochondria to the nucleus: the role of other TCA cycle enzymes

Recent studies reveal that nuclear localization of enzymes extends beyond PDH, OGDH, and FH, indicating that broader subnetworks of the TCA cycle function in mammalian nuclei. Their proximity to nuclear proteins involved in histone and nucleic acid modifications suggests “on-site” metabolite production for nuclear processes.

A recent study by Kafkia et al. revealed that a substantial portion of the TCA cycle operates within the nucleus rather than just a few isolated enzymes [69]. Using ^13^C-tracer analysis, nuclear activity was detected for glutamine-to-fumarate, citrate-to-succinate, and glutamine-to-aspartate conversions in HeLa nuclei. In mitochondria, glutamine and glutamate enter the TCA cycle via α-ketoglutarate. Given glutamine’s abundance, its nuclear availability is unlikely diffusion-limited and indeed their glutamine and citrate labeling experiments confirmed their role as nuclear TCA intermediates. Moreover, their proteomic analysis confirmed the nuclear presence of OGDH and PDH complexes, while the analysis of the Human Protein Atlas images permitted the identification of nuclear aconitase 2 (ACO2) and isocitrate dehydrogenase 3 (IDH3), which catalyze the citrate-to-α-ketoglutarate conversion. They also investigated whether the TCA cycle enzymes possess NLS. Putative NLS have been identified for nearly all steps from pyruvate to succinyl-CoA, except citrate synthase (CS). Additionally, enzymes like isocitrate dehydrogenase 1 (IDH1), succinate dehydrogenase subunits A (SDHA) and B (SDHB), and aminotransferases such as glutamate oxaloacetate transaminase 1 (GOT1) also contain NLS. Bioinformatic and proteomic analyses therefore revealed putative NLS sequences in several TCA cycle enzymes (e.g., ACO2, IDH3A, SDHA, SDHB, OGDH, SUCLG2, FH, MDH2), suggesting that these enzymes may be actively imported into the nucleus through mechanisms similar to those employed by canonical nuclear proteins. Nevertheless, the precise mechanisms governing nuclear import of TCA cycle enzymes remain unresolved. In addition to the classical importin-mediated pathway, the stimulus-dependent nature of nuclear localization observed for many of these enzymes supports a model in which nuclear entry is regulated by post-translational modifications (PTMs)—such as phosphorylation or acetylation—that either expose previously masked NLS motifs or create docking sites for importins. Moreover, proximity labeling mass spectrometry showed spatial interactions between ACO2, IDH3G, IDH1, OGDH, and nuclear proteins, further supporting their nuclear function. However, it remains an open question whether these enzymes are imported into the nucleus directly after cytoplasmic translation or whether a mechanism of mitochondrial–nuclear protein exchange also contributes to their nuclear localization. Furthermore, Kafkia and colleagues used isotope tagging (LOPIT) with succinyl-peptide enrichment in U2OS cells, revealing a high enrichment of succinylated proteins in the nucleus, particularly RNA-binding proteins. These findings align with the nuclear interactome of OGDH, suggesting localized succinyl-CoA synthesis may regulate nuclear protein succinylation [69].

Two further studies have expanded our understanding of the functional significance of nuclear translocation of TCA cycle enzymes in distinct biological contexts, including somatic cell reprogramming and chemotherapy-induced cardiac damage. First, Li W. et al. showed that ACO2, PC, CS, and IDH3A relocate to the nucleus during somatic cell reprogramming, primed-to-naive transition, and totipotency establishment, playing a key role in histone acetylation and chromatin remodeling [67]. In cardiomyocytes, nuclear translocation of enzymes like PDH-E1, malate dehydrogenase 2 (MDH-2), and isocitrate dehydrogenase 2 (IDH2) occurs in response to chemotherapy-induced mitochondrial stress, particularly anthracycline-induced cell death [65].

These findings underscore nuclear TCA enzyme translocation as a crucial mitochondria-to-nucleus communication mechanism with implications for cellular adaptation, epigenetic remodeling, and metabolic stress responses. Further research is required to define the molecular mechanisms governing nuclear TCA enzyme function and their broader impact on gene regulation and cellular homeostasis.

## Mechanisms of nuclear internalization of mitochondrial enzymes

Understanding how mitochondrial enzymes, particularly large complexes like 2-ketoacid dehydrogenases, translocate into the nucleus remains a key question in nuclear metabolic regulation. Three main hypotheses have been proposed: (1) de novo assembly in the nucleus from separately imported subunits, (2) direct translocation of fully assembled complexes via nuclear pore-dependent or independent mechanisms, and (3) specialized mitochondrial-derived vesicles (MDVs) facilitating enzyme delivery. In contrast, for other metabolic enzymes involved in nuclear acetyl-CoA production, more defined mechanisms have been described. ACSS2 translocates to the nucleus upon AMPK-mediated phosphorylation at S659, which unmasks a cryptic NLS and enables importin-dependent nuclear import, particularly under metabolic stress [46, 70]. For ACLY, several post-translational modifications have been reported to regulate its nuclear localization. AKT-mediated phosphorylation at S455 promotes nuclear acetyl-CoA production and histone acetylation [38] while acetylation of K662/665 controls ACLY nuclear import in macrophages [40]. More recently, phosphorylation at S455, T447, and S451 was shown to be critical for ACLY nuclear localization [71]. However, the precise mechanism remains less clear: these post-translational events may occur in the cytoplasm prior to nuclear import or within the nucleus itself and may either facilitate entry or stabilize ACLY once imported.

A major challenge is the size of the 2-ketoacid dehydrogenases and their lack of canonical NLS sequences. A suggestive observation was reported by Wang et al. [11], who described a putative NLS within DLST, the E2 subunit of the OGDH complex. Mutational analysis indicated that DLST is required for the nuclear distribution of OGDH, raising the possibility that this subunit contributes to nuclear targeting. However, whether DLST alone is sufficient to guide the entry of the entire complex, or whether additional mechanisms are involved, remains unclear. To date, no comparable evidence exists for PDH or other 2-ketoacid dehydrogenases. Moreover, fully assembled 2-ketoacid dehydrogenase complexes are too large for nuclear pores. PDH, for instance, is ~ 10 MDa with a 45 nm diameter, while NPCs restrict passage to molecules under 39 nm [72, 73]. The canonical PDH complex comprises a 60-subunit E2 core, 30 E1 tetramers, and 12 E3 dimers, all essential for function [74–77]. However, nuclear PDH and OGDH remain poorly characterized. Evidence suggests they may exist in smaller subcomplexes under physiological conditions [78], facilitating nuclear translocation.

Experimental studies support mitochondrial-to-nuclear translocation. Sutendra et al. examined whether nuclear PDH originates from mitochondria or is newly synthesized. Mitochondrial proteins are first translated in the ER with an N-terminal mitochondrial localization sequence (MLS), cleaved upon mitochondrial entry by mitochondrial-processing peptidase (MPP) [79, 80]. If nuclear PDH were directly translated, its precursor form should be detectable. However, only mature, cleaved PDH subunits were found in the nucleus, indicating PDH undergoes mitochondrial processing before nuclear entry. PDH-E1 knockdown reduced nuclear levels of all catalytic PDH components without affecting total cellular levels, suggesting PDH translocates as a functional complex. Importantly, no experimental evidence currently supports the alternative scenario in which PDH subunits remain in the cytosol after translation and bypass mitochondrial import before entering the nucleus; however, this possibility cannot yet be fully excluded as a potential route for nuclear PDH assembly or translocation. Additionally, nuclear PDH interacts with Hsp70, a chaperone involved in protein transport. Co-immunoprecipitation confirmed this interaction, indicating Hsp70 prevents aggregation and ensures proper folding, supporting a chaperone-mediated transport mechanism for nuclear PDH stability and function [10].

Further supporting Hsp70’s role, Mocholi et al. found that nuclear PDH-E1 levels increased upon TCR stimulation in human CD4 + T cells, independent of glycolysis. Hsp70 inhibition using KNK437 reduced nuclear PDH-E1 levels and impaired immune gene transcription, suggesting Hsp70 mediates PDH nuclear import during activation [66].

Recent findings suggest PDH may enter the nucleus through a non-canonical, NPC-independent pathway. Zervopoulos et al. proposed that PDH translocates via direct interaction with lamin A at the nuclear envelope. Electron microscopy revealed electron-dense regions at mitochondria–nucleus contact sites, indicating protein-mediated tethering facilitates enzyme transfer. Mitofusin 2 (MFN2) was identified as a key factor in enabling mitochondrial association with the nuclear envelope. Under proliferative stimuli, PDH is transported into the nucleus, retained on the lamin layer, and then released into the nucleoplasm, potentially bypassing NPC size constraints [81, 82]. However, this mechanism may be limited to proliferating cells and not occur in non-dividing cells.

Taken together, these observations suggest that nuclear PDH may not exist as a single canonical entity but rather in multiple forms. In our view, several distinct scenarios remain plausible: import of individual subunits with subsequent reassembly inside the nucleus (potentially even without prior mitochondrial import), transport of smaller subcomplexes assisted by chaperones, delivery via mitochondrial-derived vesicles, or direct transfer at mitochondria–nucleus contact sites [83]. In line with this latter possibility, recent studies have shown that cellular stress promotes the formation of nucleus-associated mitochondria (NAM), with mitochondria redistributing to the perinuclear region [83, 84]. These contact sites facilitate retrograde signaling and can mediate the direct transfer of metabolites, as exemplified by the mitochondria-derived “nuclear ATP surge” reported under confinement stress [84]. Such structures could, in principle, also facilitate the access of mitochondrial 2-ketoacid dehydrogenases to the nucleus, thereby supporting local acetyl-CoA–dependent chromatin regulation.

At present, the evidence is still limited and does not allow discrimination between these possible routes of translocation. An important open question is whether nuclear 2-ketoacid dehydrogenases function as intact holoenzymes or as modular assemblies of the three catalytic subunits, and clarifying this together with the molecular machinery mediating their import will be crucial to understand how mitochondrial metabolism is coupled to nuclear function.

## Conclusions and future perspective

Emerging evidence suggests that the nuclear localization of mitochondrial enzymes extends beyond those previously documented, such as PDH, OGDH, and FH. This finding implies that more extensive subnetworks of the TCA cycle may actively function within mammalian nuclei. In this context, additional enzymes—including ACO2, PC, CS, IDH2, and IDH3, as well as MDH2—have recently been shown to translocate to the nucleus in response to various stimuli across different cellular contexts. Future research will be essential to determine whether these enzymatic complexes remain fully assembled within the nuclear compartment or function as smaller subcomplexes. These mitochondrial enzymes are not just passive visitors; they play an active role in shaping nuclear function by locally generating metabolites essential for gene regulation and chromatin dynamics. While the production of nuclear acetyl-CoA by metabolic enzymes such as PDH, ACLY, and ACSS2 has been extensively studied—particularly concerning histone acetylation and transcriptional regulation—the broader implications of nuclear metabolic activity remain largely uncharted territory. Understanding how nuclear metabolic flux integrates with epigenetic regulation and transcriptional programs will be crucial for deciphering the full scope of mitochondria–nucleus crosstalk. An additional emerging aspect is that histone modifications themselves may function as metabolite reservoirs. Histone acetylation, beyond its classical role in transcriptional regulation, can act as a dynamic store of acetate: acetyl groups can be recycled by ACSS2 after deacetylation, redistributed between lysine residues to rapidly activate transcriptional programs, or redirected toward lipid synthesis [21, 85–90]. Similar findings have emerged for histone methylation, where lysine methylation can serve as a sink for S-adenosylmethionine (SAM), buffering the SAM/SAH ratio and thereby contributing to one-carbon metabolism independently of transcription [91, 92]. Together, these findings highlight an unexpected role of chromatin as a dynamic reservoir of metabolites, further broadening the scope of mitochondria–nucleus crosstalk.

Future studies should focus on quantifying nuclear metabolite pools, identifying additional mitochondrial enzymes with nuclear roles, and uncovering how these enzymatic activities contribute to cell fate decisions and disease processes. Additionally, structural studies will be critical for identifying chromatin interactors of these large enzymatic complexes and for elucidating how their proximity to nuclear proteins involved in histone modifications influences their functional roles. By bridging the gap between metabolism and nuclear regulation, these investigations have the potential to redefine our understanding of cellular compartmentalization and its impact on gene expression, offering a paradigm shift in how we view metabolic control in health and disease.

## Data Availability

This manuscript summarizes other studies for which data has been made available.
